# Impact of compression forces on different mesenchymal stem cell types regarding orthodontic indication

**DOI:** 10.1093/stcltm/szae057

**Published:** 2024-08-24

**Authors:** Chloé Radermacher, Rogerio B Craveiro, Wilhelm Jahnen-Dechent, Justus P Beier, Astrid Bülow, Michael Wolf, Sabine Neuss

**Affiliations:** Department of Orthodontics, University Hospital RWTH Aachen, 52074 Aachen, Germany; Helmholtz-Institute for Biomedical Engineering, Biointerface Laboratory, University Hospital RWTH Aachen, 52074 Aachen, Germany; Department of Orthodontics, University Hospital RWTH Aachen, 52074 Aachen, Germany; Helmholtz-Institute for Biomedical Engineering, Biointerface Laboratory, University Hospital RWTH Aachen, 52074 Aachen, Germany; Department for Plastic Surgery, Hand, and Burn Surgery, University Hospital RWTH Aachen, 52074 Aachen, Germany; Department for Plastic Surgery, Hand, and Burn Surgery, University Hospital RWTH Aachen, 52074 Aachen, Germany; Department of Orthodontics, University Hospital RWTH Aachen, 52074 Aachen, Germany; Helmholtz-Institute for Biomedical Engineering, Biointerface Laboratory, University Hospital RWTH Aachen, 52074 Aachen, Germany; Institute of Pathology, University Hospital RWTH Aachen, 52074 Aachen, Germany

**Keywords:** mechanobiology, stem cells, adipose-derived stem cells, bone-marrow stem cells, periodontal ligament stem cells

## Abstract

The potential of stem cells, for example upper periodontal ligament stem cells from the maxilla (u-PDLSC) and from the mandible (l-PDLSC), adipose-derived mesenchymal stem cells (AD-MSC), and bone marrow-derived mesenchymal stem cells (BM-MSC), with respect to periodontal remodeling and orthodontic treatment is of great importance. In this work, we focus on the comprehensive adaptability of different stem cell types to mechanical forces with the aim to better understanding cell behavior and to refine a new mechanistic approach to investigate periodontal remodeling. We comprehensively analyze stem cells and observe distinct morphological and proliferation changes under compression in dependence on stem cell type. The cell signaling of extracellular signal-regulated kinase (ERK) and protein kinase B, also called AKT, and their respective phosphorylation shows diverse responses to compression. Additionally, vascular endothelial growth factor and hepatocyte growth factor secretion were reduced under mechanical stress in all cell types, with cell-specific variations. Osteoprotegerin secretion was reduced under compression, particularly in u-PDLSC. At least, diverse soluble receptors of NF-kB-ligand secretion patterns among cell types under pressure were observed, providing crucial insights into bone metabolism. These findings offer a deeper understanding of the behavior of mesenchymal stem cells under mechanical stimuli, highlighting their roles in bone remodeling, wound healing, and tissue regeneration in orthodontic and regenerative medicine contexts. Our results underscore the potential of u-PDLSC, l-PDLSC, and AD-MSC in periodontal regeneration, with AD-MSC showing notable resilience under compression, indicating its promising role for further investigation for orthodontic research. While these findings are encouraging, further research is essential to fully comprehend the mechanism of stem cell-based periodontal therapies.

## Graphical Abstract

**Graphical Abstract:**
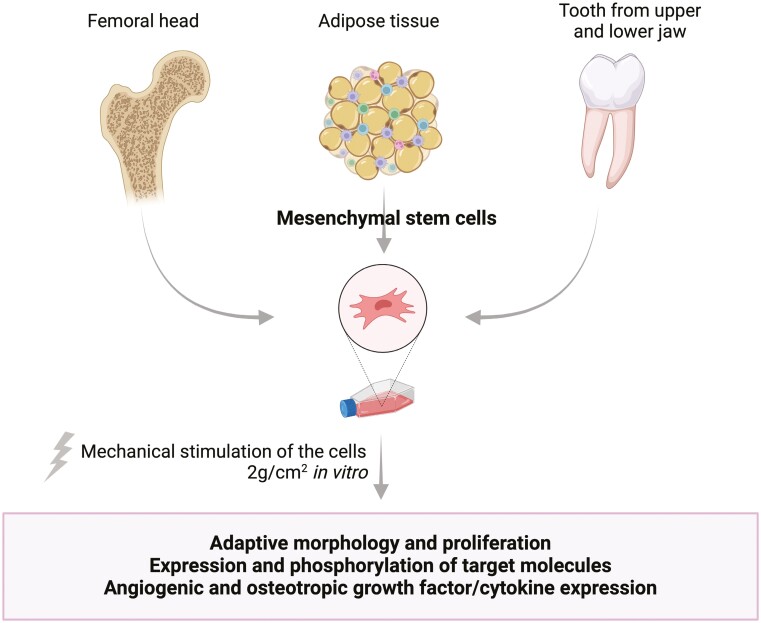

**Significance statement**

This study explores the response of various mesenchyme-derived stem cells to compression forces, often encountered in orthodontic treatments. It examines how these cells react to mechanical stress, underscoring their potential to improve regenerative therapies in orthodontics. The research is pivotal for creating more effective, customized treatments based on individual cellular responses, enhancing dental and orthodontic outcomes. Our findings provide valuable insights into integrating biomechanics with stem cell therapy, benefiting patient care.

## Introduction

Orthodontic tooth movement triggers complex biological processes in the jaw and in teeth. Mechanical force creates distinct zones of pressure and tension around the teeth. On the pressure side, the periodontal ligament (PDL) becomes compacted, which in turn triggers a cascade of cellular and biochemical changes, ultimately leading to bone resorption.^1-3^

Within this intricate process, stem cells of the PDL, termed PDLSCs, play a crucial role in periodontal tissue remodeling, due to their regenerative properties. Remarkably versatile, these cells can differentiate into a variety of cell types that are essential for repairing periodontal structures. Research has underscored their importance, showing how they contribute to the formation of new cementum and PDL in living organisms. Today, PDLSCs are investigated for their therapeutic potential in the treatment of human periodontal defects, with the initial results pointing toward positive outcomes in tissue remodeling and regeneration.^4^ Furthermore, clinical observations have indicated that periodontal tissues and alveolar bone in the maxilla (upper jaw) heal more effectively than those in the mandible (lower jaw), suggesting regional differences in the healing capabilities of these tissues. This has prompted research into the properties of PDLSCs from both regions to understand the cellular basis for these observed clinical differences.^5,6^ In addition to PDLSCs, other mesenchymal stem cell types are promising sources for periodontal regeneration, in particular adipose-derived stem cells (AD-MSCs).^7-9^ Their multi-potency and the minimally invasive procedure for their harvest make them particularly appealing.^7^ Notably, their anti-inflammatory properties may provide therapeutic benefits in addressing periodontal inflammation^10^ Despite the promising role of AD-MSCs in regenerating critical periodontal structures, the transition to clinical practice is still in progress, with ongoing efforts to overcome challenges related to standardization and verifying long-term efficacy.^7,10^ Similarly, bone marrow-derived mesenchymal stem cells (BM-MSCs) hold the potential for periodontal repair, given their capacity to differentiate into bone and other tissue types.^7^

Focusing on mechanotransduction, PDLSCs are accustomed to a sophisticated process where mechanical stress is translated into cellular responses. This ability is critical; while normal physiological compression can promote osteogenic differentiation—favorable for periodontal regeneration—excessive compression may be harmful, leading to cell death. Thus, the mechanical environment is a key factor in the effectiveness of PDLSCs in regenerative treatments.^11^ Recent progress in the application of mechanical forces has significantly enhanced the potential of AD-MSCs in the domain of dental regenerative medicine. In controlled settings, AD-MSCs demonstrate a remarkable ability to align and differentiate in response to specific mechanical stimuli. This responsiveness to mechanical cues is particularly advantageous for dental tissue remodeling thereby closely replicating the biomechanical properties of natural dental tissues. Such precision in mimicking natural tissue biomechanics is crucial for the successful reorganization, remodeling, and regeneration of dental tissues using AD-MSCs.^12^ In parallel, BM-MSCs have shown a responsiveness to mechanical compression that can be harnessed to guide their differentiation and enhance tissue repair. The full spectrum of compression effects on BM-MSCs is the subject of intense study, as researchers strive to unravel the complex interplay of factors that influence their behavior, with the goal of refining BM-MSC-based treatments for clinical application.^13-15^

The suitability of various stem cell types for cell-based therapies in the periodontal region is under investigation, with a focus on evaluating the characteristics of periodontal ligament stem cells from the upper (u-PDLSC) and lower jaw (l-PDLSCs), AD-MSCs, and BM-MSCs. The comparative analysis encompasses an assessment of each cell type's origin and its potential adaptability to the periodontal environment. This research involves monitoring the cells in *in vitro* culture conditions and assessing their response to externally applied compressive forces.

Recent studies have highlighted the significant progress made in understanding the effects of mechanical forces on various types of MSCs, particularly in the orthodontic context. For instance, one study demonstrated how mouse bone marrow mesenchymal stem cells under compressive pressure can promote osteoclastogenesis and enhance orthodontic tooth movement.^16^ In another study, researchers found that MSC transfer to the PDL during orthodontic treatment can reduce orthodontically induced root resorption and improve tooth movement.^17^ Additionally, research demonstrated that compression forces increase RANKL and decrease OPG levels in PDL cells, highlighting the importance of these cytokines in bone resorption processes during orthodontic treatments.^18^

Based on the literature, we hypothesize that mechanical compression applied to PDLSCs, AD-MSCs, and BM-MSCs will influence their behavior in distinct ways. Specifically, we predict that PDLSCs will exhibit the highest potential for promoting osteogenesis under controlled compressive forces, given their native environment's exposure to mechanical stress. In contrast, AD-MSCs, with their noted anti-inflammatory properties, may show significant improvements in periodontal tissue regeneration, particularly under lower compressive forces. BM-MSCs are expected to demonstrate a robust response to mechanical stimuli, aiding in bone and tissue repair processes.

The suitability of various stem cell types for cell-based therapies in the periodontal region is under investigation, with a focus on evaluating the characteristics of PDL stem cells from the upper (u-PDLSC) and lower jaw (l-PDLSCs), AD-MSCs, and BM-MSCs. The comparative analysis encompasses an assessment of each cell type's origin and its potential adaptability to the periodontal environment.

## Material and methods

### Cell culture

Human third molars were collected and assigned to 2 groups based on where they originated: the maxilla and the mandible. Following the previously described procedures, cells were extracted.^5^ DMEM high-glucose (Gibco), 10% fetal calf serum (FCS; Pan-Biotech), 50 mg/L L-ascorbic acid (Sigma–Aldrich), 100 IU/mL penicillin (Gibco), and 100 g/mL streptomycin (Gibco) were used to culture the isolated PDLSC. Separation was strictly enforced according to donor and origin (upper jaw or lower jaw-PDLSC = u-PDLSC and l-PDLSC) (each *n* = 3).

Adipose tissue was taken from redundant adipose tissue being excised from patients at the University Hospital RWTH Aachen Department for Plastic Surgery who were having abdominoplasty or deep inferior epigastric/muscle-sparing transverse rectus abdominis musculocutaneous flap transfer. There was both written and verbal informed consent. The regional ethics committee granted approval for this study (EK163/07), and the Declaration of Helsinki’s tenets were followed throughout the experimentation process. AD-MSC isolation was done as previously described.^19^ The cells were cultured in DMEM/F12 (Gibco), 10% FCS (Pan-Biotech), 100 IU/mL penicillin (Gibco), and 100 g/mL streptomycin (Gibco) and 10 ng/mL basic fibroblast growth factor (bFGF; Prepotech) (*n* = 3).

As previously published,^5,20,21^ BM-MSC were extracted from the bone marrow of resected femoral heads (*n* = 3) harvested during hip replacement surgery. The BM-MSC-Medium contained Mesenpan Basal Medium, 2% FCS, 1% ITS-plus (insulin, transferrin, selenious acid, bovine serum albumin, linoleic acid), 1 nM dexamethasone, 100 μM ascorbic-acid-2-phosphate, 10 ng/mL epidermal growth factor (all Pan-Biotech), 1.6 mM L-glutamine (Gibco), 80 IU/mL penicillin (Gibco), and 80 μg/mL streptomycin (Gibco) (*n* = 3).

Cells were cultured in a humidified atmosphere (20% O_2_, 5% CO_2_) at 37°C. Medium was changed every 3-4 days and all cells were tested negative for mycoplasma contamination. All experiments were performed in passage 3.

### Flow cytometry

To identify distinct mesenchyme-related surface epitopes, flow cytometry was used to examine u-PDLSC, l-PDLSC, AD-MSC, and BM-MSC. Cells were trypsinized, counted, and maintained in flow cytometry buffer (0.09% FCS in phosphate-buffered saline (Gibco)). After being spun at 500 g for 5 minutes at 4°C, the cells were resuspended in 100 mL of flow cytometry solution containing the antibodies. The flow cytometry buffer was used to dilute the APC-, PE-, or FITC-isotype controls to concentrations of 0.2 g/100 mL, 0.2 g/100 L, and 0.5 g/100 mL. Conjugated antibodies against CD34, CD45, CD73, CD90, and CD105 (eBioscience) were diluted in flow cytometry buffer at concentrations of 0.5 g/100 mL, 0.06 g/100 mL, 0.124 g/100 mL, 1 g/100 mL, and 1 g/100 mL, respectively. Following a 30-minute incubation period at 4°C, the cells were centrifuged at 500 *g* for 5 minutes to remove the supernatant. Finally, 300 μL of flow cytometry buffer was used to resuspend the cells. A minimum of 10 000 events were detected for each donor during the immunophenotype analysis using a FACS Canto II cytometer (BD Bioscience).

### Morphology and proliferation

Cells of each cell type were seeded in a density of 10 000 cells/well in 12-well culture plates in triplicates to examine proliferation. Using the Cellcyte X (CYTENA), 6 parallel, non-overlapping fields of view arranged in a 3 × 2 grids were used to picture each well. During a period of 5 days, each field of view was imaged in the “enhanced contour” (brightfield analog) once every 6 hours. Then, using Cellcyte Studio (CYTENA), confluency analysis was performed and exported for comparison.

Furthermore, the pictures taken by the Cellcyte X were used for the morphological analysis of the different cell types in different conditions. The representative pictures were all taken after 12 hours of compression.

### Compression force application

The different mesenchymal stem cell types were stimulated using an established compressive stimulation model employing sterile round glass cylinders (34 mm Ø; 18 g).^22-24^ The cells were seeded with a density of 15 000 cells/cm^2^ in 6-well plates 1 day before the compression experiment started and then mechanically stimulated over 3, 24, and 48 hours. Indeed, a static compressive force of 2 g/cm^2^ (or 0.02 N/cm^2^) was applied.

### Western blot analysis

After stimulation, immunoblotting was used to examine the cells. Cells were washed and lysed after 3, 24, and 48 hours using RIPA buffer (100 μL/well). The negative control corresponds to the uncompressed cells lysed at the same time as the compressed cells after 3 hours of loading. The amount of protein was measured using the Bradford test (Bio-Rad), and for gel electrophoresis, 25 mg of total protein was used as opposed to 10 µg for fractionated gel electrophoresis.

The protein lysates from the cells were electrophoretically separated using the 12% TGX Stain-Free FastCast (Bio-Rad), and then transferred to nitrocellulose membranes using the Trans-Blot Turbo Turbo RTA Transfer kit (Bio-Rad). Membranes were blocked in Tris-buffered saline with 0.05% Tween-20 (TBST) plus 5% BSA for 1 hour at room temperature. The primary antibodies were incubated overnight at 4°C, followed by 1 hour at room temperature with the corresponding secondary antibody. Using a ChemiDoc MP Imaging System (Bio-Rad) with Stain-Free technology and ImageLab Software (Version 6.01 Bio-Rad), immunoblot was detected by fluorescence.

### ELISA

Cell culture supernatant was collected and stored at −80°C to perform enzyme-linked immunosorbent assay (ELISA). Commercially available ELISA kits were used following manufacturers´ instructions to detect the levels of vascular endothelial growth factor (VEGF) (DVE00; R&D Systems), HGF (RAB0212; Sigma-Aldrich), OPG (TNFRSF11B; Invitrogen), and soluble receptor activator of nuclear factor kappa-Β ligand (sRANKL) (BI-20462; Biomedica).

### Statistical analysis

Graphs display the mean and SD. Using GraphPad Prism (version 10.0.2 GraphPad Prism, a 2-way analysis of variance (ANOVA) was performed, followed by a Tukeys’ post hoc test. Statistical significance was defined as a *P*-value < .05 (*P < .05; **P < .01; ***P < .001).

## Results

### Cell characterization

**Figure 1.**
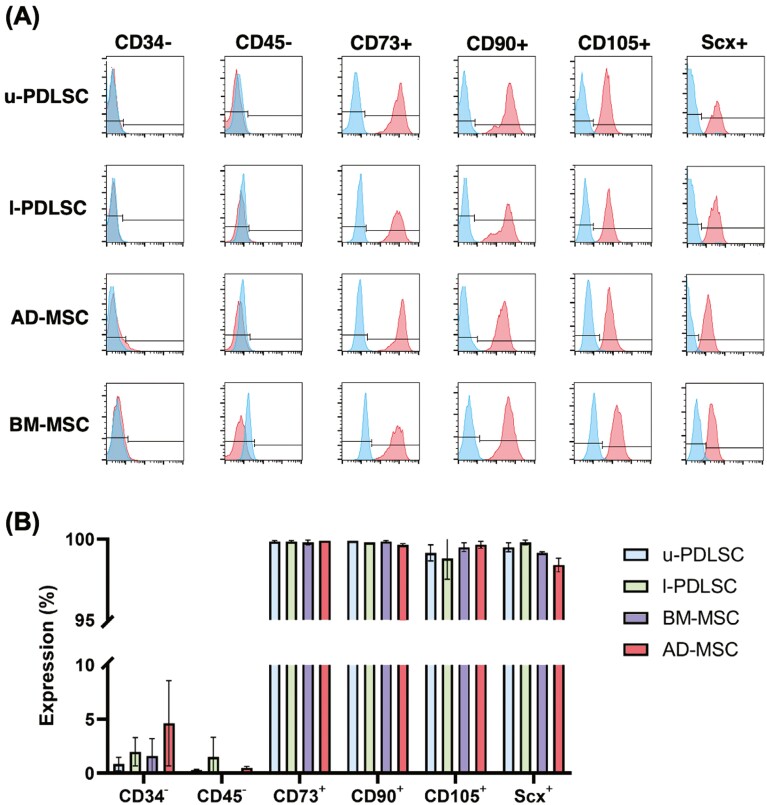
Periodontal ligament stem cells from the upper jaw (u-PDLSC) and lower jaw (l-PDLSC), AD-MSC) and BM-MSC were analyzed using flow cytometry. (A) A typical flow cytometric analysis of one specific donor’s u-PDLSC, l-PDLSC, and MSC for stem cell markers, which should stain positive (CD73+, CD90+, CD105+), as well as surface markers of hematopoietic stem cells and endothelial cells, which should stain negative (CD34−, CD45−). (B) Flow cytometric quantification of surface marker expression of the 3 donors of each cell type. CD34− and CD45− were negative for all cell types. High levels of CD73+ (> 99.8), CD90+ (> 99.8%), and CD105+ (> 98.8%) expression were detectable in all stem cell types. Abbreviations: AD-MSC, adipose-derived mesenchymal stem cells; BM-MSC, bone-marrow mesenchymal stem cells; l-PDLSC, periodontal ligament stem cells from lower jaw; u-PDLSC, periodontal ligament stem cells from the upper jaw. Biological *n* = 3

To authenticate the mesenchymal identity of the studied cell populations, a comprehensive flow cytometry analysis was conducted focusing on specific cell surface epitopes, known as characteristic for mesenchymal stem cells (MSCs) as shown in Figure 1A, B. The analysis confirmed high expression levels of characteristic MSC markers: CD73 and CD90 were expressed in over 99.6% of all cell types examined. Similarly, CD105 expression was consistently high, with a minimum recorded level of 98.8% across the cell types. In contrast, the expression of hematopoietic markers CD34 and CD45 was minimal, observed in no more than 5% of the cells, categorizing them as negative for these markers. This pattern of expression effectively validates the mesenchymal stromal cell characteristics of the analyzed populations. Additionally, the presence of Scleraxis, a transcription factor, was noted in at least 98.4% of the cells, further corroborating their stromal cell identity. Collectively, these findings demonstrate a homogenous expression profile across the cell types, with no differences in the levels of the assessed markers, thereby confirming their classification as mesenchymal stromal cells.

**Figure 2.**
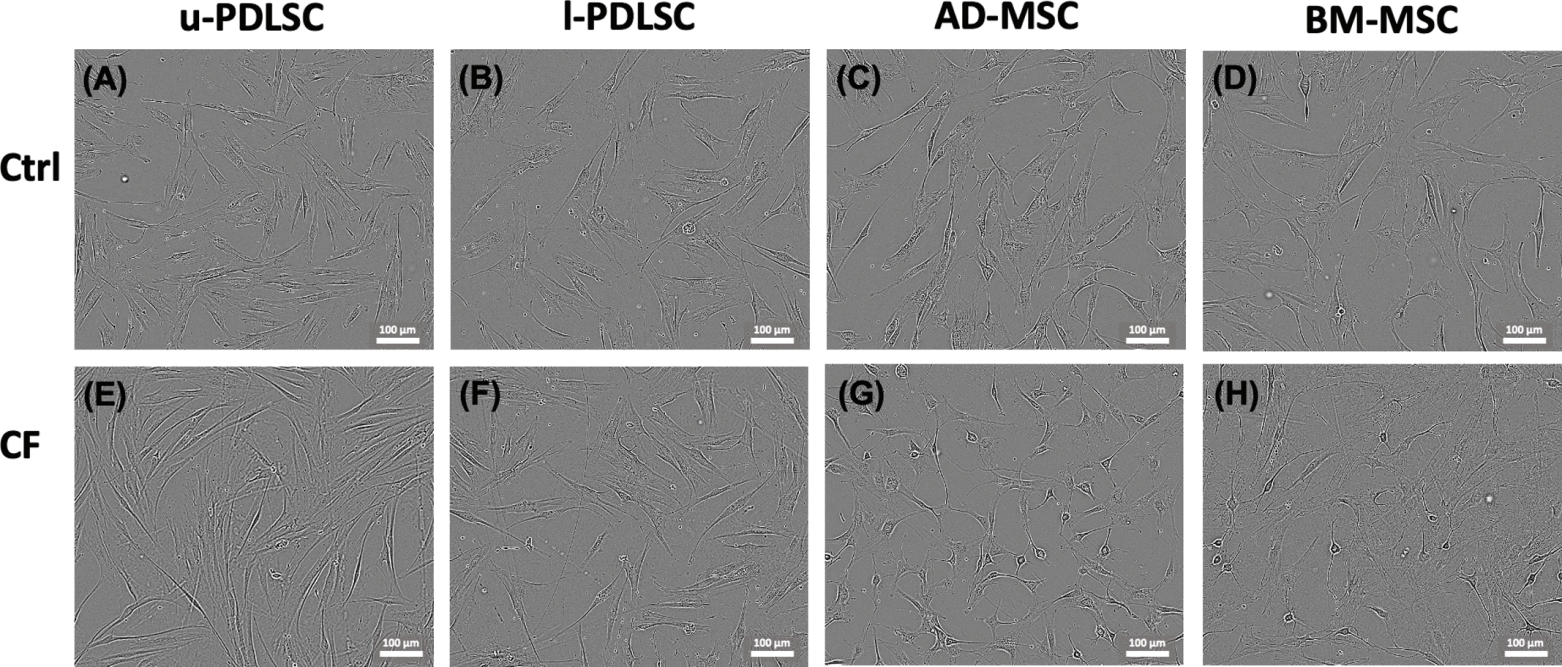
Live imaging of stromal cells using Cellcyte X “enhanced contour” mode. u-PDLSC and l-PDLSCAD-MSC, and BM-MSC were all compared morphologically in normal culture conditions (A-D) and in compressed conditions (E-H). Abbreviations: AD-MSC, adipose-derived mesenchymal stem cells; BM-MSC, bone-marrow mesenchymal stem cells; CF, compression force is applied; Ctrl, control group without compression; l-PDLSC, periodontal ligament stem cells from lower jaw; u-PDLSC, periodontal ligament stem cells from the upper jaw.

The contrast-enhance bright field microscopy revealed typical spindle-shaped, fibroblastic cell morphology of human multipotent stromal cell types (Figure 2). In the control group, the cells cultured without compression. These cells attained uniform morphology and distribution. Notably, the cytoplasm of BM-MSCs appeared more prominent when compared to other cell types, indicating a subtle variation in cytoplasmic features. Upon subjecting the cells to 12 hours of compression, u-PDLSCs and l-PDLSCs maintained their morphology, while AD-MSCs exhibited a noticeable alteration, with cells assuming a more rounded shape at the center. Similarly, BM-MSCs responded to compression by becoming noticeably flattened. These morphological changes under compression stress highlight the differential structural adaptability of various stromal cell types, which may have implications for their functional behavior under mechanical stress conditions.

### Proliferation profile under normal and compressive conditions

**Figure 3.**
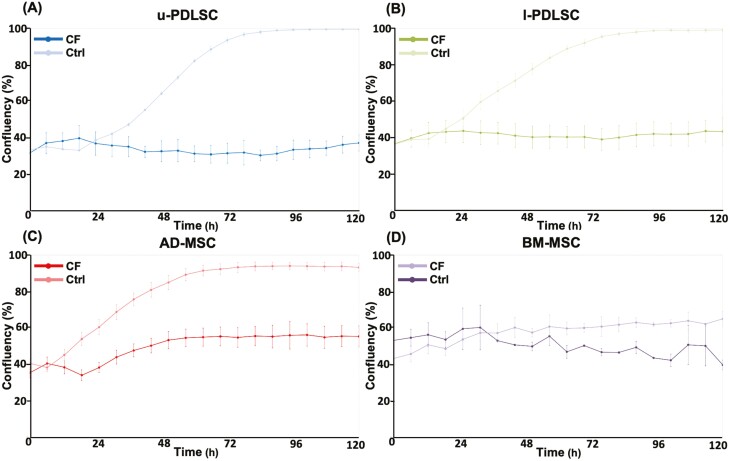
Cell proliferation analysis using using Cellcyte X live cell imaging and image analysis. Proliferation was scored as cell confluency of u-PDLSC (A), l-PDLSC (B), AD-MSC (C), and BM-MSC (D) in normal culture conditions and with compression forces over 120 hours. Abbreviations: AD-MSC, adipose-derived mesenchymal stem cells (lower left); BM-MSC, bone-marrow mesenchymal stem cells (lower right); CF, compression force is applied; Ctrl, control group without compression; l-PDLSC, periodontal ligament stem cells from lower jaw (top left); u-PDLSC, periodontal ligament stem cells from the upper jaw (top right). Biological *n* = 3 per cell type.

The impact of compressive forces on cellular proliferation is a critical aspect of understanding cellular behavior in response to mechanical stimuli, particularly in the context of orthodontic treatments. This study presents an analysis of the proliferation patterns of various cell types under compression, as illustrated in Figure 3A-D. The data reveal notable differences in the proliferation responses of u-PDLSCs, l-PDLSCs, AD-MSCs, and BM-MSCs when subjected to compressive forces. In the case of u-PDLSCs and l-PDLSCs, it is observed that uncompressed l-PDLSCs exhibit a slower proliferation rate. However, both u-PDLSCs and l-PDLSCs respond similarly to compression, showing a slight increase in cell confluence after 24 hours. This increase is transient and reverses by 48 hours, after which the confluency stabilizes. The observed increase in cell confluency under compression may be attributed to the more flattened morphology of these cells.

For AD-MSCs, the proliferation curve in the absence of compression parallels that of the l-PDLSCs during the logarithmic growth phase. Surprisingly, compressed AD-MSCs demonstrated a unique response among the studied cell types, exhibiting a significant increase in cell confluency after 24 hours. BM-MSCs generally proliferated markedly slower than all other cell types. Under compressive force, BM-MSCs stopped proliferating altogether, suggesting that mechanical stimulation directed the cells into the quiescent state or even triggered cell death. This finding underscores the differential responses of mesenchymal stem cell types to mechanical stress, highlighting the complexity of cellular behavior in orthodontic contexts.

### Expression and phosphorylation of targets molecules under compressive forces

**Figure 4.**
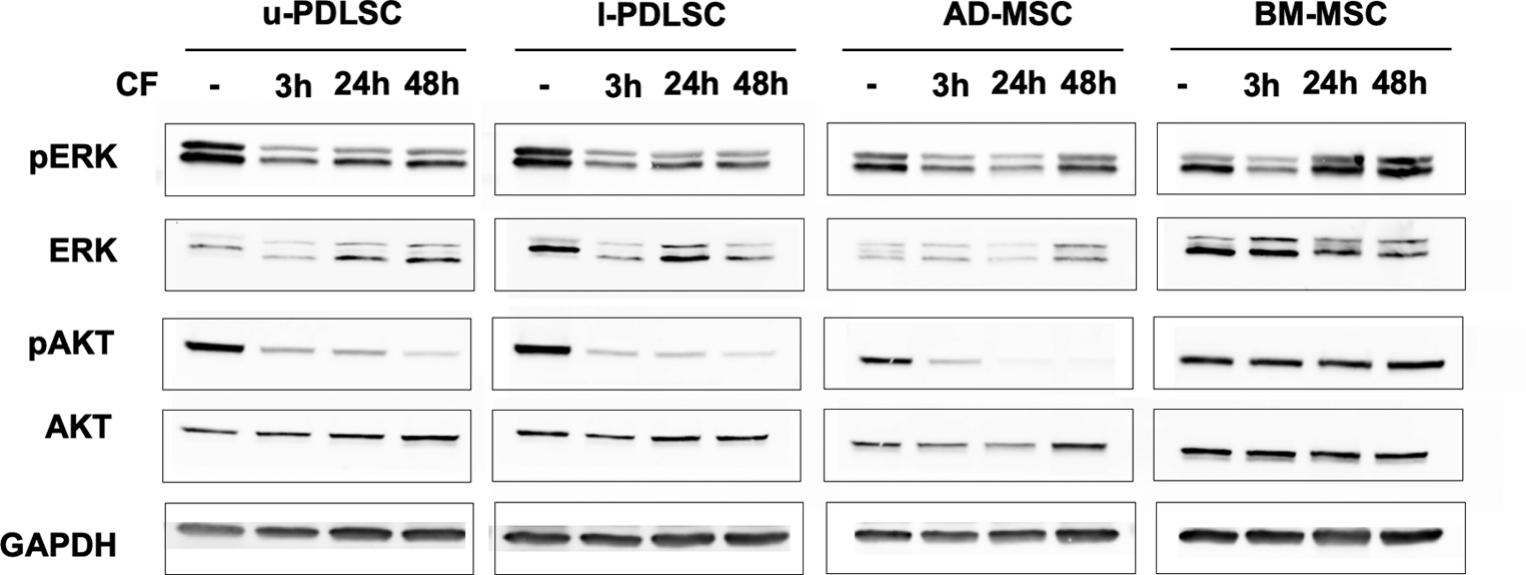
Protein expression analysis of cellular kinase ERK and AKT. Cell extracts were prepared from u-PDLSC, l-PDLSC, AD-MSC, and BM-MSC in static culture or following compressive force applied for 3, 24, and 48 hours. Extracts were separated by SDS-PAGE, blotted onto nitrocellulose membrane and probed with antibodies directed against whole ERK and AKT or phosphoERK (pERK) and phosphoAKT (pAKT), respectively. Abbreviations: AD-MSC, adipose-derived mesenchymal stem cells; BM-MSC, bone-marrow mesenchymal stem cells; CF, compression force is applied; l-PDLSC, periodontal ligament stem cells from lower jaw; u-PDLSC, periodontal ligament stem cells from the upper jaw. Biological *n* = 3 per cell type.

The study of signal transducing kinase expression levels with and without treatment conditions informs cellular responses in orthodontic treatments. Figure 4 illustrates expression levels of the proliferation-associated kinases ERK and AKT, which were analyzed by Western blot using antibodies against total ERK and AKT, and phosphoforms phosphoERK and phosphoAKT, respectively. The findings reveal distinct expression patterns for these kinases in PDLSCs, AD-MSCs, BM-MSCs under varying compressive forces.

In the absence of compressive forces, phosphoERK expression is notably higher in PDLSCs compared to AD-MSCs and BM-MSCs. Upon 3 hours of stimulation, all cell types exhibit a reduction in phosphoERK levels, with a slight increase observed in u-PDLSCs and l-PDLSCs, a significant elevation in BM-MSCs, and a subsequent decrease in AD-MSCs after 24 hours. At the 48-hour mark, phosphoERK levels stabilize in u-PDLSCs, l-PDLSCs, and BM-MSCs, while AD-MSCs show an increased expression.

Conversely, the unphosphorylated ERK shows minimal expression in u-PDLSCs and AD-MSCs, with higher levels in l-PDLSCs and BM-MSCs when not subjected to compressive forces. The expression of ERK remains unchanged in u-PDLSCs, AD-MSCs, and BM-MSCs, but decreases in l-PDLSCs after 3 hours. After 24 hours, there is an upregulation in u-PDLSCs and l-PDLSCs, while a downregulation is observed in the other cell types. At the final time point, ERK expression stabilizes in u-PDLSCs and BM-MSCs, decreases in l-PDLSCs, and increases in AD-MSCs.

The expression profile of phosphoAKT over time is similar among u-PDLSCs, l-PDLSCs, and AD-MSCs. The control groups of all 4 cell types, along with compressed BM-MSCs, display comparable expression levels. However, compressive forces lead to a decreased expression in u-PDLSCs, l-PDLSCs, and AD-MSCs. Between 3 and 24 hours, the expression remains stable in u-PDLSCs and l-PDLSCs but decreases subsequently. AD-MSCs show a marked downregulation starting from 24 hours. In contrast, BM-MSCs maintain a stable AKT expression level over time, regardless of stimulation. In AD-MSCs, compressive forces initially induce a gradual downregulation until 24 hours, followed by a significant increase in expression after 48 hours.

### Angiogenic and osteotropic growth factor/cytokine expression

**Figure 5.**
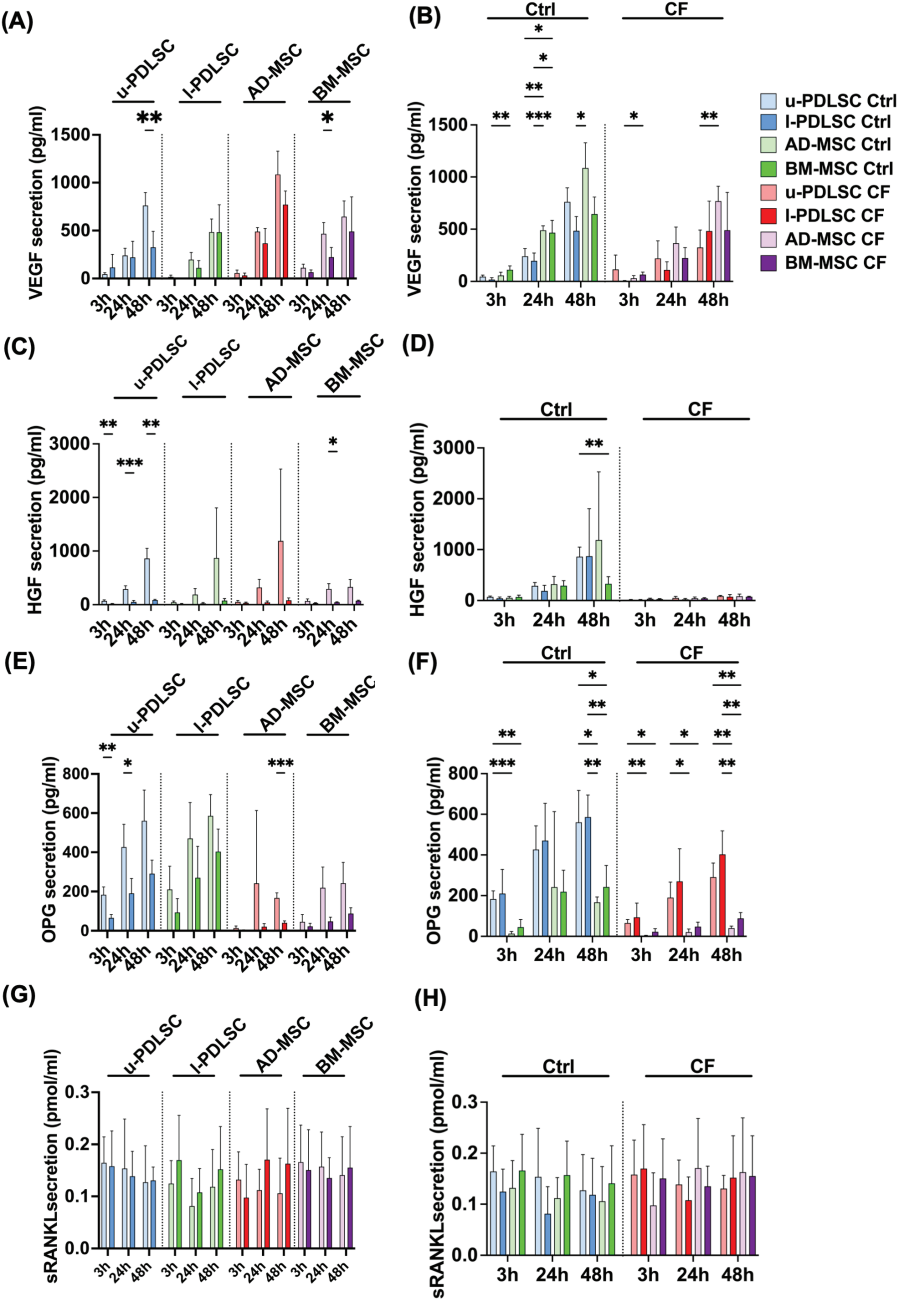
Angiogenic and osteogenic growth factor/cytokine expression. Vascular endothelial growth factor (VEGF, A-B), hepatocyte growth factor (HGF, C-D), osteoprotegerin (OPG E-F), and soluble receptor of NF-κB-Ligand (sRANKL, G-H) were measured by ELISA in the supernatant of u-PDLSC, l-PDLSC, AD-MSC, and BM-MSC with or without compressive force applied. On the left side: pairwise comparison of the respective levels with and without compression within each cell type at each time point. On the right side: comparison of the respective levels between the different cell types in normal culture conditions and with compressive force application. Data were analyzed by 2-way analysis of variance (ANOVA) followed by Tukeys’ post hoc test was performed. Abbreviations: ADMSC, adipose-derived mesenchymal stem cells; BM-MSC, bone-marrow mesenchymal stem cells; CF, compression force is applied; Ctrl, control group without compression; l-PDLSC, periodontal ligament stem cells from lower jaw; u-PDLSC, periodontal ligament stem cells from the upper jaw. Statistically significant differences to control are marked by asterisks (*P < .05; **P < .01; *** *P* < .001). Biological *n* = 3 per cell type.

The secretion of VEGF and of hepatocyte growth factor (HGF)/scatter factor, both regulating wound healing and angiogenesis was measured. Figure 5A-B illustrates the impact of cellular compression on VEGF secretion in u-PDLSCs, l-PDLSCs, BM-MSCs, and AD-MSCs. VEGF secretion was significantly reduced in u-PDLSCs at 48 hours post-compression. The expression was roughly halved (57.3%) compared to static culture, indicating a pronounced response to mechanical stress. Likewise, BM-MSCs decreased their VEGF secretion as early as 24 hours post-compression, with a 52.1% reduction compared to the control. Comparative analysis of VEGF secretion across different cell types 24 hours post-compression revealed that both u-PDLSCs and l-PDLSCs secreted significantly lower amounts of VEGF compared to AD-MSCs and BM-MSCs. Specifically, u-PDLSCs had 48.1% reduced, and l-PDLSCs had 57.8% reduced VEGF secretion post-compression. After 48 hours of static culture, cells secreted highly variable amounts of VEGF with AD-MSCs secreting more than twice the amount VEGF (+124.2%) compared to l-PDLCs. This disparity highlights the inherent differences in VEGF secretion capabilities among these cell types.

Under compressive force, AD-MSCs secreted significantly more VEGF than l-PDLSCs at both 3 and 48 hours. At 3 hours, AD-MSCs show an 8-fold, and at 48 hours, a 1.6% increase is observed. These results suggest a robust VEGF response in AD-MSCs under mechanical stress conditions.

Hepatocyte growth factor (HGF) has beneficial effects in wound healing and tissue regeneration processes (eg, it is pro-angiogenic, anti-fibrotic) and its secretion dynamics offer valuable insights into cell behavior under varied conditions. Figure 5C shows that HGF secretion differed slightly between cell types with or without mechanical stimulation. Overall, compressive stimulation inhibited HGF secretion, particularly in u-PDLSCs at all measured time points and in BM-MSCs after 24 hours. In u-PDLSCs under compressive stress, there was a pronounced decrease in HGF secretion compared to the control group, with reductions of approximately 76.4% after 3 hours, 82.1% after 24 hours, and 90.0% after 48 hours. This demonstrates a consistent and significant inhibition of HGF secretion following mechanical stimulation. Mechanically stimulated BM-MSCs reduced their HGF expression by 84.7% at 24 hours. Moreover, a significant difference is observed in the control groups at the 48-hour mark, where u-PDLSCs exhibit HGF secretion at a rate more than 161.8% higher than BM-MSCs, highlighting a substantial differential in HGF production between these cell types. Additionally, as depicted in Figure 5D, there is a tendency for BM-MSCs to secrete less HGF compared to other cell types. This pattern emphasizes the cell-specific responses to mechanical stimuli, particularly in the context of HGF secretion, and underscores the importance of understanding cellular behavior in response to varying environmental conditions.

Osteoprotegerin (OPG) is a soluble decoy receptor antagonizing the cytokine, sRANKL. Together these proteins regulate bone remodeling. We measured secretion of OPG and sRANKL using ELISA. Figure 5E-F illustrates that OPG secretion was inhibited in compressed cells. Notably, a significant reduction in OPG levels was observed in u-PDLSCs at both 24 and 48 hours, with a decrease of approximately 55.3% and 48.1%, respectively, compared to their control counterparts. Similarly, AD-MSCs showed a significant decrease in OPG secretion at 48 hours under compression, with a reduction of approximately 75.8%. When comparing different cell types, it was found that u-PDLSCs, both with and without stimulation, secreted significantly higher levels of OPG compared to AD-MSCs and BM-MSCs. The difference was particularly pronounced in the absence of compression, where u-PDLSCs showed an OPG secretion rate that was approximately 1236.5% and 303.3% higher than that of AD-MSCs and BM-MSCs, respectively. Even under compression, u-PDLSCs maintained a higher secretion rate, being approximately 2330.8% and 192.4% higher than AD-MSCs and BM-MSCs, respectively. However, after 48 hours, no significant differences in OPG secretion were observed between uncompressed and compressed l-PDLSCs. This data underscores the differential response of various mesenchymal stem cell types to mechanical stimulation and their potential role in bone remodeling processes.

Soluble receptor activator of nuclear factor kappa-Β ligand (sRANKL) was likewise measured to assess its secretion under mechanical stimulation. Figure 5G-H shows that sRANKL secretion responded to mechanical stimulation. U-PDLSCs and AD-MSCs exhibited a tendency to increase sRANKL concentration whereas, in contrast, BM-MSCs and l-PDLSCs initially showed a decrease in sRANKL levels at 3 and 24 hours, followed by a reversal of this trend. Interestingly, in the absence of pressure, AD-MSCs were observed to secrete sRANKL at higher levels on average compared to u-PDLSCs, with BM-MSCs and l-PDLSCs following in sequence. This pattern, however, reversed at the 48-hour mark. Under conditions of mechanical stress, no significant differences in sRANKL secretion were noted among the cell types. These observations provide valuable insights into the cell-specific responses to mechanical stimuli and their potential implications in bone remodeling and pathophysiology.

## Discussion

The distinct responses of MSCs from different tissues to mechanical compression observed in our study suggest intrinsic adaptations to their native environments. For example, adipose-derived MSCs exhibit notable resilience to mechanical stresses, likely reflecting the conditions prevalent in adipose tissue. These observations underscore the need for further investigations to determine whether such properties are inherent to MSCs or primarily acquired during *in vitro* culture. Employing advanced culture techniques that more closely mimic the dynamic *in vivo* environment may be essential to distinguish between these possibilities.

However, cell-based therapies, especially those including (MSCs), are highly promising for enhancing regenerative approaches in dental medicine. Such therapies facilitate tissue regeneration, enhance the integration of implants, and provide innovative reparative options for dental and periodontal conditions. Notably, MSCs are recognized as superior candidates for tissue replacement treatments, particularly in regenerating alveolar bone and managing periodontal diseases.^25^ Clinical research has confirmed the efficacy of stem cells for bone restoration, periodontitis management, and the regeneration of dental pulp. These investigations underscore the therapeutic advantages and safety of stem cells obtained from dental tissues.^26^

In this study, mesenchymal stromal cells (MSCs) are characterized by their plastic-adherence, their fibroblastic morphology and expression of CD105, CD73, and CD90, and their lack of CD45 and CD34 expression, along with the ability to differentiate into osteoblasts, adipocytes, and chondroblasts in vitro, as established in key studies.^27^ In the initial phase of this study, the identity of MSCs was confirmed by flow cytometric analysis of these expression markers, complemented by a detailed morphological assessment. As described previously,^27^ all mesenchymal stem cell types typically exhibit a fibroblast-like morphology, characterized by a spindle-shaped appearance.

Subsequent proliferation analysis revealed that u-PDLSCs exhibit a more rapid growth rate compared to l-PDLSCs and AD-MSCs. In contrast, BM-MSCs demonstrated a relatively slower proliferation rate under standard culture conditions. This confirms the previously published comparison between the proliferation of u-PDLSC, l-PDLSC, and BM-MSC.^5^ However, when subjected to compressive forces, both u-PDLSCs and l-PDLSCs display similar responses, whereas AD-MSCs exhibit a notably enhanced proliferation rate. In strong contrast, BM-MSCs appear incapable of withstanding the application of such forces. No similar culture conditions were applied to AD-MSCs or BM-MSCs earlier, whereas it is a standard model to mimic orthodontic compression forces and therefore applied on periodontium-related cells.^28^ Different from previous proliferation analysis under compression force^29^ is that, through live imaging, the cells are not loosened by manipulation before analysis which makes it a more accurate method.

In terms of protein expression, phosphoERK, ERK, phosphoAKT, and AKT exhibit analogous patterns in u-PDLSCs, l-PDLSCs, and AD-MSCs. Conversely, BM-MSCs show no discernible response to the application of pressure. Research indicates that modulating the ERK pathway in PDLSCs can influence the balance between osteogenic differentiation and osteoclastogenesis, thereby impacting the efficiency and outcomes of orthodontic treatments.^30,31^ The activation of phosphoERK in PDLSCs under mechanical stress regulates key cellular behaviors, including proliferation, differentiation, and apoptosis, essential for periodontal tissue remodeling during orthodontic tooth movement.^32,33^ Moreover, phosphoAKT plays a critical role in regulating various cellular functions in PDLSCs, including cell survival, proliferation, and differentiation, which are key to tissue remodeling during orthodontic tooth movement.^32^ Studies suggest that targeting the AKT pathway in PDLSCs can influence orthodontic treatment outcomes by affecting processes like osteoblastogenesis and alveolar bone remodeling, highlighting its potential therapeutic significance.^34^

To understand the differential responses of MSCs to compressive forces, it is imperative to consider the unique mechanotransduction pathways and cellular mechanisms inherent to each cell type. The distinct morphological and proliferative adaptations observed in u-PDLSCs, l-PDLSCs, AD-MSCs, and BM-MSCs under compression may be reflective of their intrinsic mechanical thresholds and resilience. For instance, the robust proliferation of AD-MSCs under compressive stress could be attributed to their heightened sensitivity to mechanotransduction signals, which may activate pathways conducive to cell survival and proliferation. Conversely, the susceptibility of BM-MSCs to stress-induced quiescence or apoptosis could indicate a lower mechanical tolerance, possibly due to differences in cytoskeletal organization or expression of mechanoreceptors. The modulation of signaling pathways such as ERK and AKT, pivotal in cell survival, proliferation, and differentiation, further underscores the cell-type-specific responses to mechanical stimuli. These observations suggest that the capacity for orthodontic tissue remodeling and regeneration may be intricately linked to the cell’s ability to interpret and respond to mechanical cues within its microenvironment. Integrating these insights, future research should aim to delineate the molecular underpinnings of such differential responses, potentially unlocking targeted therapeutic strategies to harness the regenerative capabilities of these cells in orthodontic and regenerative medicine.

Furthermore, VEGF secretion is predominantly observed in uncompressed AD-MSCs, with compressive forces leading to a significant reduction in VEGF secretion exclusively in u-PDLSCs. Research indicates that enhancing VEGF secretion could be a therapeutic strategy to improve periodontal regeneration and accelerate orthodontic tooth movement by promoting better vascular support and tissue repair.^30,32,34^

However, HGF secretion is inhibited by compressive forces across all 4 examined cell types at every time point. Notably, the basal level of HGF secretion in uncompressed conditions is lowest in BM-MSCs. The available scientific literature does not provide direct evidence or specific studies focusing on the role of hepatocyte growth factor (HGF) in periodontal remodeling, especially in the context of orthodontic tooth movement or PDL cell dynamics. HGF is known for its roles in cell growth, cell motility, and morphogenesis in various tissues.^20,35^ Moreover, HGF stimulates MSC proliferation, a key process in tissue regeneration and repair, making it vital for therapeutic applications in regenerative medicine.^20,36^

The balance between sRANKL and osteoprotegerin (OPG) plays a critical role in periodontal remodeling, particularly in the regulation of bone resorption and formation.^37^ sRANKL promotes osteoclast differentiation and activation, leading to bone resorption, which is a key process in the remodeling of alveolar bone during orthodontic treatment and periodontal disease progression.^38^ Conversely, OPG acts as a decoy receptor for sRANKL, inhibiting its interaction with RANK on osteoclasts, thereby reducing bone resorption and helping to maintain bone homeostasis in the periodontium.^39^ Here, the concentration of Osteoprotegerin (OPG) is found to be higher in PDLSCs, particularly in l-PDLSCs, as compared to AD-MSCs and BM-MSCs in the supernatant of non-compressed cells, with a reduction observed upon compression. Intriguingly, sRANKL does not exhibit significant variations across the conditions tested.

The differential activation of signal transduction pathways, such as ERK and AKT, plays a crucial role in how these cells interpret and respond to mechanical stimuli. For instance, studies have shown that the ERK pathway can significantly influence osteogenic differentiation by mediating cellular responses to mechanical stress, which is particularly relevant for the behavior of PDLSCs under compressive forces. Similarly, the AKT pathway, known for its role in promoting cell survival and proliferation, could explain the robustness of AD-MSCs in resisting mechanical stress and their potential in regenerative therapies.^30^

Beyond the specific context of orthodontics, these findings have profound implications for the design of biomaterials and therapeutic strategies across various fields of regenerative medicine. Understanding the mechanotransduction pathways in MSCs can guide the development of engineered tissues and scaffolds that mimic natural mechanical environments, enhancing tissue integration and functionality. For example, incorporating mechanical cues into scaffold designs could improve the regenerative capacities of implanted materials by aligning stem cell responses with desired therapeutic outcomes.^40^ Therefore, this study not only sheds light on the cellular mechanics of MSCs under compression but also sets the stage for future innovations in biomaterials and regenerative therapies that leverage these mechanobiological insights. Further research in this direction could potentially revolutionize how we approach tissue engineering and regenerative medicine, offering more effective and tailored treatment options for a variety of conditions.^41^

## Conclusion

This study sheds light on the diverse responses of MSCs to mechanical compression, highlighting the nuanced roles of u-PDLSCs, l-PDLSCs, AD-MSCs, and BM-MSCs in periodontal remodeling and regeneration. The resilience of AD-MSCs to compressive forces, juxtaposed with the vulnerability of BM-MSCs, underscores the complexity of cell-specific mechanotransduction mechanisms and their implications for orthodontic treatment strategies. These findings not only reinforce the potential of utilizing MSCs in periodontal regeneration but also beckon further investigation into the molecular pathways governing cell behavior under mechanical stress. Future studies elucidating these mechanisms are essential for the development of optimized cell-based therapies, aimed at enhancing periodontal regeneration and improving outcomes in orthodontic treatments. This research marks a step forward in our understanding of stem cell dynamics in orthodontic contexts, paving the way for novel regenerative approaches tailored to the mechanobiological intricacies of periodontal tissue.

## Data Availability

The data underlying this article will be shared on reasonable request to the corresponding author.
